# Fracture Load and Fracture Patterns of Monolithic Three-Unit Anterior Fixed Dental Prostheses after In Vitro Artificial Aging—A Comparison between Color-Gradient and Strength-Gradient Multilayer Zirconia Materials with Varying Yttria Content

**DOI:** 10.3390/jcm11174982

**Published:** 2022-08-25

**Authors:** Max L. Pöppel, Martin Rosentritt, Richard Sturm, Florian Beuer, Jeremias Hey, Alois Schmid, Franziska Schmidt

**Affiliations:** 1 Department of Prosthodontics, Geriatric Dentistry and Craniomadibular Disorders, Charité Universitätsmedizin Berlin, Aßmannshauser Str. 4-6, 14197 Berlin, Germany; 2 Department of Prosthodontics, University of Regensburg, Franz-Josef-Strauss-Allee 11, 93053 Regensburg, Germany; 3 Department of Dental Preservation, Preventive and Paediatric Dentistry, Charité Universitätsmedizin Berlin, Aßmannshauser Str. 4-6, 14197 Berlin, Germany

**Keywords:** zirconia, multilayer, fracture load, fractography, roughness, finite element modelling

## Abstract

(1) Background: Due to advantages such as avoidance of chipping, pulp-friendly tooth preparation and cost reduction, zirconia is increasingly being used monolithically without veneering. Nevertheless, to enable good aesthetics, various multilayer systems have been developed. The aim of this study was to investigate the impact of different zirconia multilayer strategies and yttria levels on fracture load, fracture pattern, stress distribution and surface roughness. (2) Methods: Monolithic three-unit anterior FDPs were made from three different color-gradient zirconia multilayer materials with different yttria levels corresponding to varying strength and degrees of translucency grades (Katana HTML, STML, UTML, Kuraray) and one strength-gradient zirconia multilayer material (Katana YML, Kuraray) and artificially aged in a chewing simulator (1.2 × 10^6^ load cycles, 50 N, 2 × 3000 thermocycles, 5–55 °C). Analyses of fracture load, fracture pattern, fracture surfaces, stress distribution and roughness were performed after the fracture load test. Shapiro–Wilk, Kruskal–Wallis, Mann–Whitney U-tests and one-way ANOVA were used (*p* < 0.05). (3) Results: Fracture loads of the high strength color-gradient material HTML and the strength-gradient material YML were comparable after 5 years of aging (*p* = 0.645). Increasing yttria levels resulted in a decrease in fracture resistance of 42–57% (*p* < 0.05). Surface roughness of different zirconia generations is comparable after polishing and aging. (4) Conclusions: Color-gradient multilayer zirconia materials and new strength-gradient zirconia materials with similar yttria levels in the basal layers show comparable mechanical properties and are suitable for anterior FDPs.

## 1. Introduction

In recent years, zirconia (ZrO_2_) has developed from a uniform material into a diverse family of materials with different optical and mechanical properties [1,2]. The first two zirconia generations doped with 3 mol% yttria are mainly composed of tetragonal crystals. They are characterized by superior flexural strength (1000–1500 MPa) and high fracture toughness (3.5–4.5 MPa√m) [3]. These properties are due to the small grain size (~0.3–0.6 µm) [4,5,6] and transformation toughening [7]. This results from the conversion of the metastable tetragonal grains to monoclinic grains and is accompanied by a 3–5% increase in volume (martensitic transformation). This thereby leads to compressive stress at crack tips and prevents further crack propagation [2,3,8]. The first and second generation differ mainly in their alumina (Al_2_O_3_) content. The high strength in the first zirconia generations comes along with low translucency. The high opacity is caused by the optical anisotropic behavior of the tetragonal crystals, which lead to strong birefringence and light scattering effects at the grain boundaries [3]. Therefore, they are often veneered with silicate ceramics to meet the patient’s demand for esthetic restorations. The veneering, however, is susceptible to fractures (chippings) [9]. Several efforts have been made to overcome the chipping problems. The most effective strategy to avoid chipping is to use monolithic designs [9]. In addition to the reduced chipping problem, monolithic restorations require less invasive tooth preparation [10] and allow for cost- and time-efficient fabrication using CAD/CAM [1]. Due to these advantages, further zirconia types for monolithic restorations with optimized optical properties have been developed. In the third generation zirconia, the yttria content was increased to 5 mol% [1,2,3,7]. This creates an additional cubic phase characterized by an optical isotropic behavior of the crystals and a larger grain size (0.7–4 µm) [3,5,6,7], which reduces the number of grain boundaries and increases translucency [11]. The cubic phase is not capable of undergoing phase transformation [5]. Therefore, flexural strength and fracture toughness of the third generation (400–900 MPa, 2.2–2.7 MPa√m) are comparable to those of lithium disilicate [3,12]. The fourth generation, which is 4 mol% yttria stabilized, tries to balance mechanical (600–1000 MPa, 2.5–3.5 MPa√m) and optical properties [3]. To enable the application of monolithic restorations in the visible area, multilayer materials with color- and translucency gradients that imitate the natural appearance of teeth were developed. It is possible that the layered manufacturing process of the zirconia blanks affects the mechanical properties. All zirconia generations are represented in the Katana zirconia multilayer system by the Japanese manufacturer Kuraray Noritake. Kaizer et al. found flexural strength values of 800–900 MPa for a second generation multilayer zirconia material (Katana Zirconia HTML), 560–650 MPa for a fourth generation multilayer zirconia material (Katana Zirconia STML) and 470–500 MPa for a third generation multilayer zirconia material (Katana Zirconia UTML) [13].

It was feared that monolithic zirconia restorations were harmful to the natural tooth structure of the antagonist teeth due to their great hardness [7,14,15]. Studies have shown that when the surface is very smooth, the wear of zirconia is very low [12] and the wear of the antagonist is similar or even lower than that of silicate ceramics [15,16]. Surface treatment and roughness are more decisive for wear than surface hardness [17].

Another problem with monolithic zirconia restorations is the unprotected contact of the material with the moist oral cavity. There is a risk of so-called low-temperature degradation (LTD). This is a gradual transformation from the tetragonal to the monoclinic phase in the presence of water [18,19]. However, for 3Y-TZP, it has been shown that this does not negatively affect the fracture strength, since the transition is limited to the surface (transformation depth 5–60 µm) [4,7,15,18,19]. The third and fourth generation zirconia show little or no phase transformation [4,5,7,14]. With them, only a lower fatigue strength of the material has been observed so far [8,20]. 

In chewing simulation, the restoration is simultaneously exposed to thermal and mechanical loads in a humid environment. Repeated cyclic loading well below the loading limits can lead to the development and the progression of internal or external defects. The remaining stability is measured by a subsequent fracture test. The stability of zirconia is determined by the interaction of the material fatigue through subcritical crack growth and transformation toughening.

We have extensive knowledge of the material properties of the various zirconia generations with constant yttria contents. Recently, zirconia with different yttria content in one material and resulting strength gradients has been offered. It is unknown which mechanical properties result from the combination. Although any efforts to increase translucency are particularly important in the anterior region, the effects of mechanical loading on monolithic anterior FDPs made of zirconia have hardly been investigated. The purpose of this study is to compare the mechanical properties of monolithic three-unit anterior FDPs made of color-gradient materials, which have been on the market for a few years, with a new color- and strength-gradient material after aging.

The null hypotheses are:

- there is no difference in the fracture load of monolithic three-unit anterior FDPs made of color-gradient zirconia materials and those made of color- and strength-gradient zirconia materials after aging;
- there is no difference in fracture type, fracture origin and stress distribution between color-gradient and strength-gradient FDPs;
- due to the different grain sizes, different zirconia materials exhibit different roughness values after polishing and aging.

## 2. Materials and Methods

### 2.1. Materials, Specimens Manufacturing, Aging Procedure and Fracture Load Test

STL files of a monolithic three-unit anterior FDP and two abutments (right upper central incisor, right upper canine) were designed by a CAD/CAM software (Meshmixer and Fusion360, Autodesk, Mill Valley, CA, USA). The abutments had a 1 mm deep circumferential shoulder finish line, a 6 degrees convergence angle, a preparation height of 6 mm, rounded inner angles and were milled of base metal CoCr alloy (ZirLux, Henry Schein Inc., Melville, LA, USA). The restoration had a minimum wall thickness of 1 mm, a triangular shaped connector with gingival base, a connector cross-sectional area of 23 mm^2^ mesial and 21.5 mm^2^ distal and an inter abutment span of 7 mm. A comparable FDP geometry with similar cross sections is described in the literature [21].

**Figure 1:**
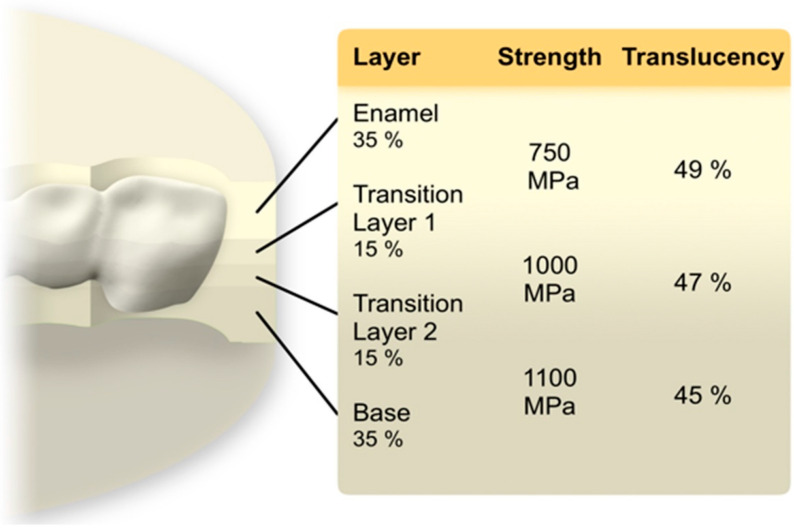
Multilayer architecture of a YML zirconia blank and nesting position. Data regarding the layer thickness, strength and translucency values was supplied by the manufacturer Kuraray Noritake.

The FDP was enlarged 22.5–22.7% to compensate for sinter shrinkage. Three color-gradient zirconia materials with different strength and translucency levels were employed in this study: high strength zirconia material Katana Zirconia HTML (Katana Zirconia High translucency multi layered, Kuraray Noritake, Hattersheim, Germany), medium strength material Katana Zirconia STML (Super translucent multi layered, Kuraray Noritake), and low strength high translucency zirconia Katana Zirconia UTML (Ultra translucent multi layered, Kuraray Noritake). Each blank consisted of 4 different layers (35% Enamel layer, 15% Transition layer 1, 15% Transition layer 2, 35% body layer). In addition, one color- and strength-gradient zirconia material YML (Yttria multi layered, Kuraray Noritake) was used, where the Enamel layer is composed of STML, the transition layers 1 and 2 as well as the body layer consists of a new type of zirconia with strength values > 1000 MPa (Figure 1) [22]. The FDPs were nested in the middle of the zirconia blanks. The specimens (*n* = 8/group) were dry milled (white stage zirconia blanks, shade A2, T 18 mm, ∅ 98.5 mm) by a five-axis laboratory milling machine (PrograMill PM7, Ivoclar Vivadent AG, Schaan, Liechtenstein) and sintered to full density with a conventional 7 h sintering program according to the manufacturer’s recommendations (HT-S speed, MIHM Vogt GmbH, Stutensee, Germany). The FDPs were polished with a goat hairbrush (15,000 rpm) and a diamond polishing paste (Zirkopol, Feguramed, Buchen, Germany). Inner surfaces and the abutments were air-particle abraded (Al_2_O_3_, 50 µm, 0.2 MPa, 10 s, 15 mm) and ultrasonically cleaned for 5 min in 96% ethanol. After drying a primer was applied on both surfaces (Clearfil ceramic primer plus, Kuraray Noritake). The FPDs were cemented using a dual cure luting resin (Panavia V5, Kuraray Noritake). Excess was removed with a foam pellet and then the compound was light cured 10s per surface (385–515 nm, 1000 mW/cm^2^) (LED curing light Valo Grand, Ultradent, South Jordan, UT, USA). To simulate the periodontal ligament the abutments were movably embedded in a 135° angle using cold curing resin (PalaXpress, Kulzer, Hanau, Germany) and polyether impression material (Impregum penta soft, 3M ESPE, Landsberg am Lech, Germany). In a first step, the abutments were dipped in melted wax, resulting in a 0.8 ± 0.1 mm thick wax layer. In a second step after embedding, the wax was completely removed and replaced with polyether [23].

**Figure 2:**
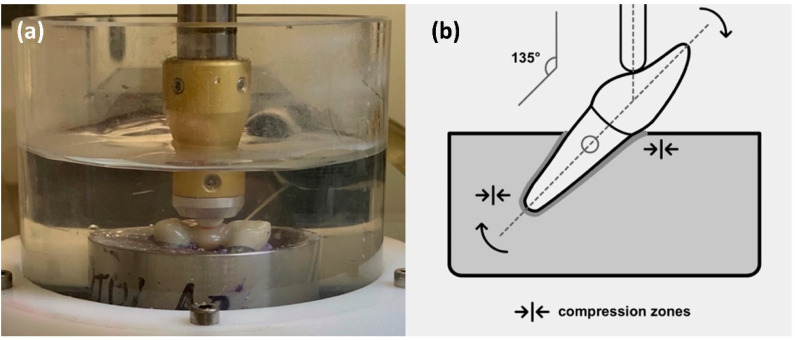
(**a**) Test set-up in the chewing simulator; (**b**) scheme for the test set-up.

All specimens were subjected to artificial aging. First, they were stored in distilled water for 24 h at 37 °C (Incubator B28, Binder Tuttlingen, Germany). Mechanical loading (1.2 × 10^6^ cycles, 50 N, 1.93 Hz, descendant speed 40 mm/s, ascendant speed 60 mm/s, mouth opening 2 mm, steatite antagonist ∅ 6 mm, loading point middle of the pontic) and thermal cycling (2 × 3000 cycles, 5–55°C, 90 s dwell time, 30 s drain time, distilled water) was performed simultaneously using a chewing simulator (CS-4, SD Mechatronic, Feldkirchen-Westerham, Germany) (Figure 2).

Fracture load (FL) was determined using a universal testing machine (Zwick Z010, ZwickRoell, Ulm, Germany) with a crosshead speed of 1 mm/min. A 0.3 mm tin foil (Dentaurum, Ispringen, Germany) was placed between the FDPs and the semi-spherical stainless-steel indenter (∅ 5 mm) to avoid stress peaks. The machine was stopped at a 20% force drop off. FL was defined as maximum force before a sudden decrease in the load-deformation curve, which was associated with visible damage and a typical cracking sound.

### 2.2. Fracture Pattern Analysis and Fractography

All specimens were examined under a digital light microscope (VHX-5000, Keyence, Osaka, Japan) after manufacturing, aging and fracture load test. Photos of the fracture patterns and surfaces at different magnifications (20–100×) as well as 3D scans were made. Fracture locations were noted. A fractographic analysis to determine the fracture origin was performed. The evaluation was made according to established standards [24].

### 2.3. Finite Element Method

The finite element method (FEM) was used to analyze the stress distribution under load (Fusion360, Autodesk). The finite element model consisted of 69,583 elements and 115,412 nodes. Material properties of zirconia (E-modulus 210 GPa, Poisson’s ratio 0.30), CoCr alloy (240 GPa, 0.25), luting resin (6.3 GPa, 0.30) and embedding material (2.3 GPa, 0.49) were taken from manufacturer’s data sheets. Linear elasticity and isotropy in behavior with no residual stresses from sintering, air-particle abrasion and luting were assumed. A load of 450 N was simulated. The results were shown in color graphs, the same color visualized the same stress level.

### 2.4. Mean Roughness Values

The mean roughness value (R_a_) was determined to evaluate the surface roughness. It was examined with a white light profilometer (Infinite focus G4, Alicona imaging, Graz, Austria) after polishing and aging. The roughness values were measured at a suitable area without significant curvature on the palatinal surface close to the loading point. The following device settings were used: objective with 50× magnification, a vertical resolution of 41 nm, a lateral resolution of 2.14 µm and a contrast value of 1.36. The investigated area was 284 × 215.5 µm. The Lc-value was set to 150 µm to reduce environmental noise.

### 2.5. Statistical Analysis

For data evaluation descriptive statistics were performed (SPSS 27, IBM Corp., Armonk, NY, USA). Shapiro–Wilk test checked the normal distribution of the results. For the fracture load non-parametric tests (Kruskal–Wallis test, Mann–Whitney U test) were utilized to compare different groups. To detect differences for roughness values between the groups one-way analysis of variance (one-way ANOVA) was performed. *p*-values < 0.05 were defined as significant.

## 3. Results

### 3.1. Fracture Load

**Figure 3:**
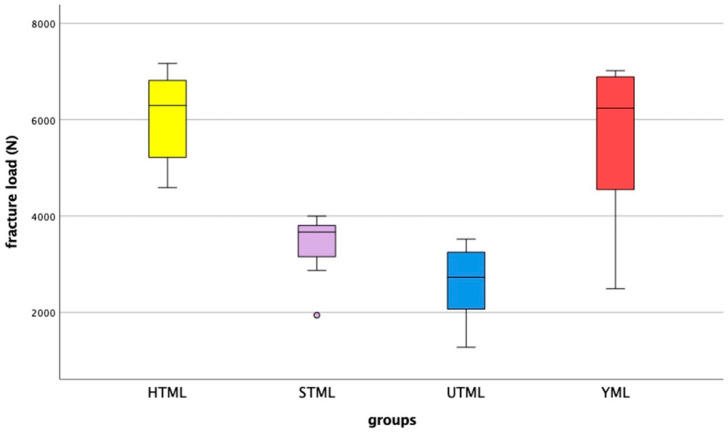
Fracture load results after artificial aging by chewing simulation, showing median, interquartile range (IQR, 75th percentile (Q1), 25th percentile (Q3)), upper whisker (Q1–1.5 IQR), lower whisker (Q3–1.5 IQR), and outliers.

All FDPs survived chewing simulation without failure or chipping. Values between 1274 N and 7169 N were measured for the fracture loads of the specimens (Figure 3). Table 1 shows the values for the different multilayer zirconia materials. Shapiro–Wilk testing indicated that not all results were normally distributed, therefore non-parametric tests were utilized to compare the results. Kruskal-Wallis testing suggested that there were significant differences between the groups (*p* < 0.001). Multiple Mann-Whitney U tests proved that all groups showed significant differences from each other, except for HTML and YML (*p* = 0.645).

### 3.2. Fracture Pattern and Fractography

**Figure 4:**
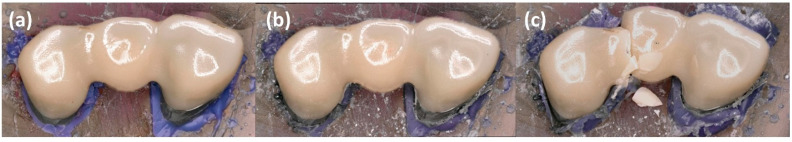
Representative specimen after (**a**) manufacturing, (**b**) aging and (**c**) fracture load test.

**Figure 5:**
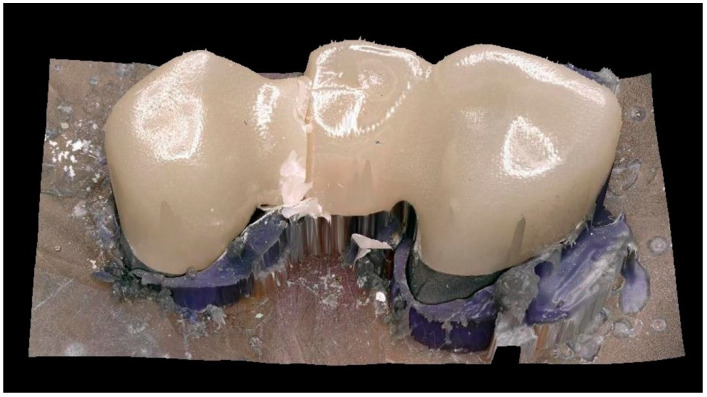
3D Scan of a fracture pattern.

After aging, no or only minimal wear was visible on the surface at 50× magnification (Figure 4). The FDPs showed brittle fractures. For STML and YML, 100% of the FDP’s fractured at the distal connector, while for HTML the figure was 87.5% (Figure 5). UTML showed more diverse fracture patterns and various fracture points at the connectors (50% distal connector, 25% mesial connector, 25% other locations).

**Figure 6:**
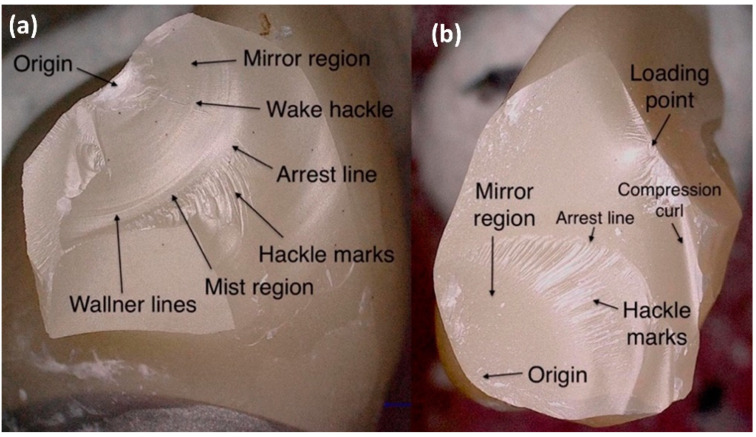
Fractographic analysis: (**a**) fracture origin near the loading point. This fracture type usually occurs with HTML and YML; (**b**) fracture origin on the gingival side of the pontic. This fracture type usually occurs with STML and UTML.

In the examination of the fracture surfaces, all specimens showed damage at the loading point. For HTML and YML the fracture origin was mainly located near the loading point. With STML and UTML specimens, the origin was on the gingival side of the connector or there were signs of fracture origin at the loading point as well as on the basal side. Origin defects were surrounded by a smooth semielliptical mirror region. Fine hackle marks radiating outwards indicated the direction of crack propagation. Some specimens showed typical compression curls on the loading side (Figure 6).

### 3.3. Finite Element Method 

**Figure 7:**
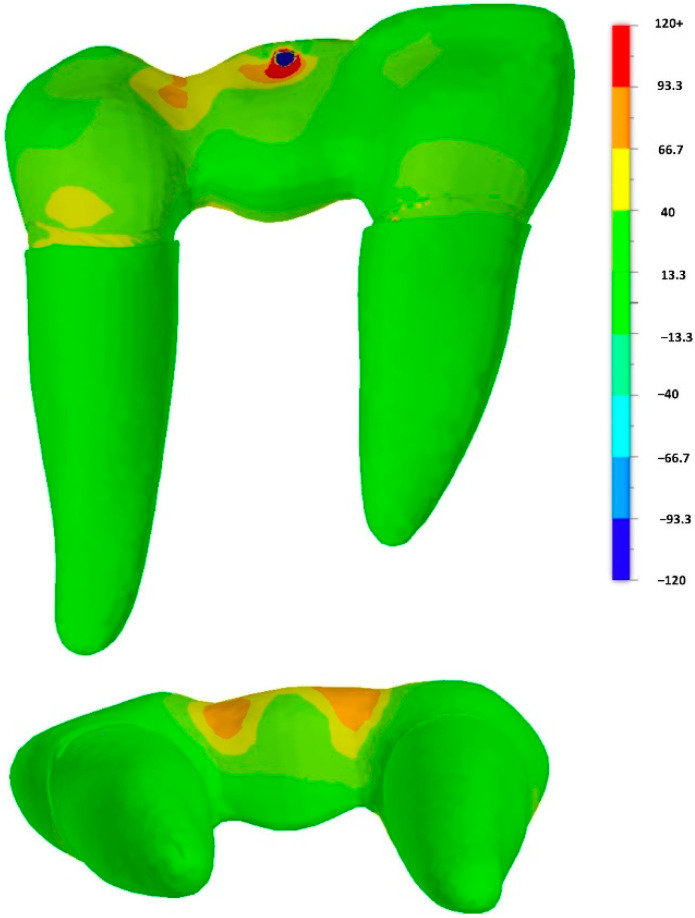
FE analysis: principal stress [MPa] distribution within the restoration (central occlusal load direction).

The FEM analysis showed that a stress peak occurred at the loading point. The simulation confirmed high stress concentration on the distal connector. FEM analysis indicated high tensile stress on the gingival connector side, especially in the vestibular area. Other areas showed only minor loads (Figure 7).

### 3.4. Mean Roughness Values

**Figure 8:**
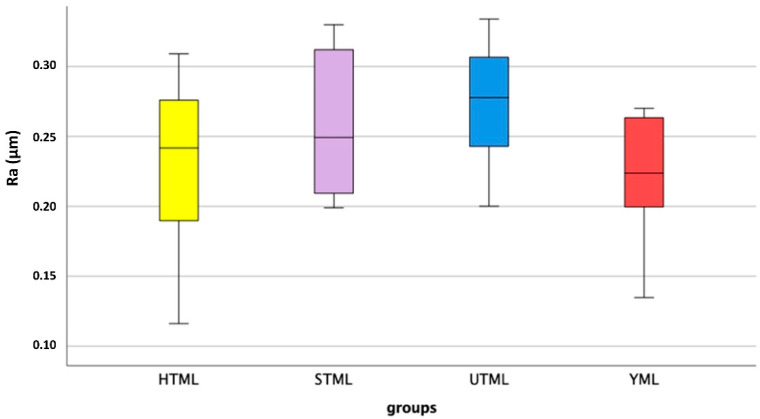
Roughness values R_a_ (µm) after aging: median, interquartile range (IQR, 75th percentile (Q1), 25th percentile (Q3)), upper whisker (Q1–1.5 IQR), lower whisker (Q3–1.5 IQR).

**Figure 9:**
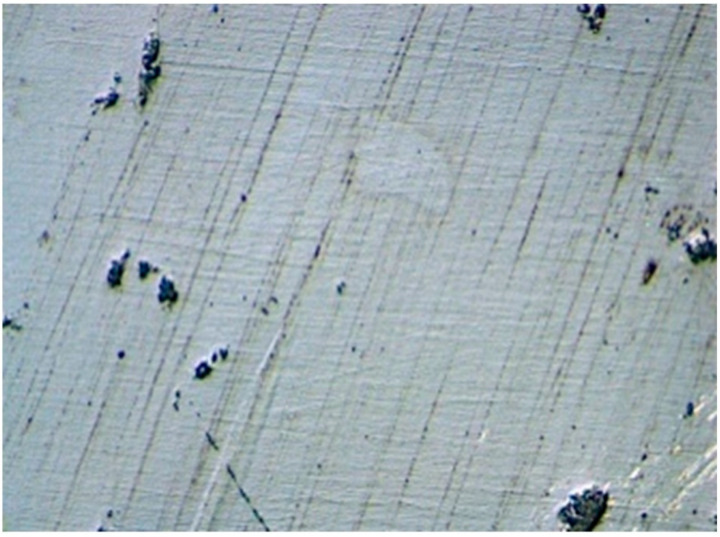
Zirconia surface with fine grooves parallel to the polishing direction.

The surfaces showed a smooth surface structure with fine grooves parallel to the direction of polishing and a few pits. The roughness measurements showed similar roughness R_a_ values for the four materials (*p* = 0.197; One-way ANOVA) (Figure 8 and Figure 9). The mean, maximum and minimum values are listed in Table 2.

## 4. Discussion

### 4.1. Influence of Multilayer Architecture to Fracture Strength, Fracture Pattern, Fracture Origin and Stress Distribution

There was very little scientific literature on monolithic anterior FDPs made of zirconia and none of them focused on the load-bearing capacity of different multilayer systems [21,25]. The first hypothesis, that there is no difference in fracture load of color-gradient and strength-gradient zirconia materials, must be rejected partially. While the color-gradient material HTML and the strength-gradient material YML showed comparable results, there were major differences within the group of color-gradient materials. Consistent with previous studies, the results proved that the fracture resistance is highly dependent on the yttria level [12,21,26]. The higher the yttria content, the lower the mechanical properties [4,8,27]. Other studies showed that in addition to the yttria content, layer thickness and connector cross-sectional area are the decisive factors for strength [10,25,27,28,29,30,31]. Connector height is more important than connector width [31]. The same minimal layer thickness does not apply to all zirconia materials [27]. While it is well documented that a layer thickness of 0.5 mm is sufficient for 3Y-TZP to withstand chewing forces [10,29], the minimum layer thickness for 4Y-PSZ and 5Y-PSZ is controversial. It seems that zirconia with smaller restoration thickness and cubic zirconia benefits more from adhesive luting, while the cement type has hardly any influence on the fracture load of thicker or veneered zirconia restorations [29,32,33]. The manufacturer’s recommendations should be strictly followed, especially because STML and UTML did not show any transformation toughening [5,7,8]. The material 5Y-PSZ occupies an intermediate position between conventional zirconia and lithium disilicate (LiSi_2_). It has lower translucency and a higher flexural strength than LiSi_2_ [11,16,34]. After adhesive cementation, the two materials showed comparable fracture loads, because LiSi_2_ benefits more from adhesive luting [12,32]. However, thinner walls are possible with zirconia [10,32] and zirconia causes less wear at the restoration and the opposing teeth [15], while 4Y-PSZ is a trade-off between strength and aesthetics. 

Color-gradient multilayer materials contain the same yttria level in all layers. Therefore, theoretically, the layers do not differ in their mechanical properties. Different esthetics result from different concentrations of metal oxides for pigmentation [1,5]. Compared to monolayer architecture, color-gradient multilayer architecture showed no disadvantages in terms of load-bearing capacity [7,12,14]. Strength-gradient multilayer blanks contain different generations of zirconia in different layers [1,34,35,36]. Therefore, the layers differ in their properties. According to the manufacturer, the top layer of YML contains the same material as STML and the base layers are comparable to HTML. In the case of this hybrid zirconia material, there are concerns that different sinter shrinkage of the layers leads to internal stresses and thus impairs the long-term reliability. However, Michalova et al. proved that a strength-gradient material can be less affected by artificial aging than a color-gradient material [34]. Schönhoff et al. confirmed comparable Weibull moduli of different layers after dynamic fatigue aging [36]. For materials with a strength-gradient, the nesting of the restoration in the blank must be considered. The connectors of FDPs should be placed in the 3Y-TZP area [35]. Moreover, the possibility that there are differences between the layers also exists with the color-gradient type. Harada et al. showed slightly different shrinkage rates of different color layers [4], Wille et al. proved differences in susceptibility to LTD between enamel and transition layers [18], and Kaizer et al. showed that cross-sectional multilayer specimens had a lower fracture resistance than homogeneous beams [13]. All of this indicates weaknesses in the intermediate layers due to the layered manufacturing process and an influence of coloring oxides. In addition, slightly different yttria concentrations in different layers of multilayer materials marketed as color-gradients were found [4]. However, Kolakarnprasert et al. could not find any differences in yttria level and microstructure between the layers of the Katana color-gradient materials [5]. Top layer and base layer showed comparable flexural strength [13]. The susceptibility to aging of the layers varies greatly depending on the manufacturer [18]. This shows that there is no sharp boundary between color-gradient and strength-gradient materials. The properties of these multilayer blanks are highly dependent on different manufacturing processes used by different manufacturers. Elsayed et al. found comparable or even higher fracture load values (7530 N (3Y-TZP), 5000 N (4Y-PSZ), 3700 N (5Y-PSZ)) for crowns with similar test setup (CoCr dies, simulated 5 years aging) [26]. Michailova et al. found values of 3535 N for a 4Y-PSZ color-gradient material and 5040 N for a 3/5Y-PSZ strength-gradient material [34]. Zacher et al.’s investigation of anterior FDPs on implants resulted in lower fracture load values (2094 N (4Y-PSZ), 1269 N (5Y-PSZ)) [21]. Rosentritt et al. determined a mean fracture load of 1760N for posterior FDPs made of a 3/5Y-PSZ strength-gradient material on human teeth [35]. The comparability of the results of different studies is limited due to the large number of influencing factors (restoration geometry, chewing simulation parameters, abutment material, periodontal ligament simulation).

According to ISO6872:2015 HTML and YML are class 5 ceramics, STML is a class 4 and UTML a class 3 material. Class 3 ceramics have minimum flexural strength requirements of 300 MPa and they are suitable for three-unit FDPs not involving the molar region. Class 4 (>500 MPa) and 5 (>800 MPa) are suitable for posterior FDPs. During chewing simulation, none of the tested FDPs cracked or chipped. After aging, the remaining fracture load was greater than 1000 N for all zirconia materials. Chewing forces around 1000 N are described for bruxism in the posterior region. In the front tooth area, the forces may be smaller. Therefore, all multilayer zirconia materials were suitable for anterior three-unit FDPs.

The second hypotheses must be partially rejected, because there were differences in fracture patterns between UTML and the other groups. The higher the yttria level, the more catastrophic and varied the fracture patterns. In addition, as the yttria content increased, there was a clear shift in fracture origin to the gingival side of the connector. The 5Y-PSZ material was more prone to tensile loads than 3Y-TZP. In accordance with other studies, all specimens survived mastication simulation [21,35]. The fractures in the universal testing machine mainly occurred at the connector with the smallest cross-sectional area. This is in accordance with previous studies [21,25]. Fracture origins at the loading point [37] as well as on the tensile side of the connector are described in the literature [21,25,35]. In accordance with the findings of Heintze et al., FEM analysis showed stress concentration near the loading point and at the basal side of the connector [31]. Hackle marks and fracture mirrors indicated high energy and velocity fractures [24,36].

### 4.2. Influence of the Yttria Level to Mean Roughness Values

The next hypothesis, that different zirconia generations show significant different roughness levels after aging, must be rejected. There were no significant differences in the surface roughness values of the different groups. However, materials with higher yttria proportion tended to be rougher than materials with lower yttria content. This can be explained by different grain sizes of the materials [8]. UTML (1.7–4.05 µm) and STML (0.68–2.8 µm) have a larger grain size than HTML (0.55–0.62 µm) [5,6]. Grain-pullouts that occurred during the polishing process or during thermo-mechanical aging led to different surface roughening. This is consistent with the literature in that 5Y-PSZ is slightly less smooth than 3Y-TZP [38]. Thermocycling and LTD roughen the surface of zirconia [39,40]. In turn, rough zirconia is more susceptible to hydrothermal aging [40]. Other negative consequences of rough surfaces such as plaque accumulation followed by caries and periodontal inflammation, more chipping at veneered restorations, external staining and excessive wear of the antagonist might be theoretical risks. Our study showed that surface roughness of the different generations is comparable with this polishing protocol after artificial aging. The fact that other studies showed comparable restoration and antagonist wear of all generations, which are lower than that of lithium disilicate, supports our findings [15,16,34]. Furthermore, surface hardness and modulus of elasticity are hardly influenced by the change of the yttria content [4,7,14]. Monolithic zirconia is smoother than lithium disilicate or layered zirconia [15,38,39]. Glazed zirconia restorations showed the least surface roughness [38], but the long-term durability of the glaze is questionable [17]. After the glaze is lost due to occlusal adjustment or wear, monolithic zirconia can be polished back nearly to the level of glazed zirconia [38,40]. This is not possible for glass ceramics [38]. The value range of our results between 0.2–0.3 µm agrees with Caglar et al. (0.28 µm) for polishing Katana HT with zirconia polishers [41]. Our values are very close to the threshold (R_a_ = 0.2 µm) below the roughness has no further impact on bacterial colonization [42]. The tongue can perceive roughness values between 0.25–0.5 µm [14]. Linkevicius et al. showed that significantly lower roughness values down to 0.05 µm are possible for monolithic zirconia by using multiple polishing steps [43].

A limitation of this study is that only materials of one manufacturer were tested. The deviation of the modulus of elasticity of the artificial alloy abutments from the modulus of elasticity of the teeth and the large connector cross-section may have led to higher fracture loads. Due to the high rigidity of the metal dies, the critical tensile loads are reduced in comparison to natural teeth or polymer abutments. This can lead to an overestimation of the resistance of the tested materials [44,45]. In addition, the polyether coating of the roots cannot mimic the complex behavior of the natural periodontal ligament.

## 5. Conclusions

Clinicians should keep in mind the following points:

- all tested color-gradient and strength-gradient multilayer zirconia materials are suitable for monolithic three-unit anterior FDPs;
- the new strength-gradient multilayer material Katana YML shows comparable load-bearing capacity to the color-gradient multilayer material Katana HTML;
- in color-gradient zirconia materials, increasing the yttria content to improve translucency leads to a reduction in the fracture load of 42% (STML) and 57% (UTML) in comparison to HTML. Therefore, the indication should be carefully considered;
- the weak points in FDPs are the connectors.

## Figures and Tables

**Table 1:** Fracture load values (FL) in N after artificial aging by chewing simulation.

Material *	*n*	Median	Maximum	Minimum	Interquartile Range
HTML (3Y-TZP)	8	6296	7169	4588	5176–6918
STML (4Y-PSZ)	8	3668	3998	1940	3011–3837
UTML (5Y-PSZ)	8	2726	3521	1274	2041–3272
YML (3–4Y-PSZ)	8	6239	7018	2493	4505–6907

* TZP: tetragonal zirconia polycrystal, PSZ: partly stabilized zirconia.

**Table 2:** Roughness values R_a_ and R_z_ after aging.

		R_a_ (µm)				R_z_ (µm)			
Material *	*n*	Mean	Max.	Min.	SD	Mean	Max.	Min.	SD
HTML (3Y-TZP)	8	0.230	0.309	0.116	0.064	2.016	3.003	0.850	0.667
STML (4Y-PSZ)	8	0.259	0.330	0.199	0.053	2.099	3.358	1.447	0.604
UTML (5Y-PSZ)	8	0.274	0.334	0.200	0.046	1.946	2.351	1.357	0.334
YML (3–4Y-PSZ)	8	0.222	0.270	0.135	0.046	2.010	3.258	1.119	0.652

* TZP: tetragonal zirconia polycrystal, PSZ: partly stabilized zirconia.

## Data Availability

Not applicable.
